# Stereotactic radiosurgery vs. fractionated radiotherapy for tumor control in vestibular schwannoma patients: a systematic review

**DOI:** 10.1007/s00701-017-3164-6

**Published:** 2017-04-13

**Authors:** Oscar Persson, Jiri Bartek, Netanel Ben Shalom, Theresa Wangerid, Asgeir Store Jakola, Petter Förander

**Affiliations:** 1 Department of Neurosurgery R03:02, Karolinska University Hospital, 171 76 Stockholm, Sweden; 2 Department of Clinical Neuroscience, Karolinska Institutet, Stockholm, Sweden; 3 Department of Neurosurgery, Copenhagen University Hospital Rigshospitalet, Copenhagen, Denmark; 4 Department of Neurosurgery, Rabin Medical Center, Petah Tikva, Israel; 5 Department of Neurology, St; Göran Hospital, Stockholm, Sweden; 6 Department of Neurosurgery, St. Olavs Hospital, 7006 Trondheim, Norway; 7 Department of Neurosurgery, Sahlgrenska University Hospital, Blå Stråket 5, vån 3, 41345 Göteborg, Sweden; 8 Department of Clinical Neuroscience, Institute of Neuroscience and Physiology, Sahlgrenska Academy, Box 430, 40530 Göteborg, Sweden

**Keywords:** Vestibular schwannoma, Stereotactic radiosurgery, Fractionated stereotactic radiotherapy, Gamma Knife, LINAC

## Abstract

**Objective:**

Repeated controlled studies have revealed that stereotactic radiosurgery is better than microsurgery for patients with vestibular schwannoma (VS) <3 cm in need of intervention. In this systematic review we aimed to compare results from single-fraction stereotactic radiosurgery (SRS) to fractionated stereotactic radiotherapy (FSRT) for patients with VS.

**Data sources and eligibility criteria:**

We systematically searched MEDLINE, Web of Science, Embase and Cochrane and screened relevant articles for references. Publications from 1995 through 2014 with a minimum of 50 adult (>18 years) patients with unilateral VS, followed for a median of >5 years, were eligible for inclusion. After screening titles and abstracts of the 1094 identified articles and systematically reviewing 98 of these articles, 19 were included.

**Intervention:**

Patients with unilateral VS treated with radiosurgery were compared to patients treated with fractionated stereotactic radiotherapy.

**Results:**

No randomized controlled trial (RCT) was identified. None of the identified controlled studies comparing SRS with FSRT were eligible according to the inclusion criteria. Nineteen case series on SRS (*n* = 17) and FSRT (*n* = 2) were included in the systematic review. Loss of tumor control necessitating a new VS-targeted intervention was found in an average of 5.0% of the patients treated with SRS and in 4.8% treated with FSRT. Mean deterioration ratio for patients with serviceable hearing before treatment was 49% for SRS and 45% for FSRT, respectively. The risk for facial nerve deterioration was 3.6% for SRS and 11.2% for FSRT and for trigeminal nerve deterioration 6.0% for SRS and 8.4% for FSRT. Since these results were obtained from case series, a regular meta-analysis was not attempted.

**Conclusion:**

SRS and FSRT are both noninvasive treatment alternatives for patients with VS with low rates of treatment failure in need of rescue therapy. In this selection of patients, the progression-free survival rates were on the order of 92–100% for both treatment options. There is a lack of high-quality studies comparing radiation therapy alternatives for patients with VS. Finally, 19 articles reported long-term tumor control after SRS, while only 2 articles reported long-term FSRT results, making effect estimates more uncertain for FSRT.

**Electronic supplementary material:**

The online version of this article (doi:10.1007/s00701-017-3164-6) contains supplementary material, which is available to authorized users.

## Introduction

Vestibular schwannomas (VSs) are benign intracranial tumors arising from the Schwann cells of the eight cranial nerve. The incidence has been reported to be approximately 2 in 100,000 [21, 27, 31, 49]. Although benign, these tumors have the ability to grow and can cause significant symptoms due to compression of the cerebellum or brain stem or impairment of the vestibulocochlear nerve function. Once the VS is discovered, symptoms like unilateral sensorineural hearing loss, vertigo and tinnitus can usually be traced back several years [37].

Three different management strategies are commonly applied after a diagnosis of VS. Conservative management including regular scheduled magnetic resonance imaging (MRI) and audiometry is frequently used for small asymptomatic tumors. If the VS show signs of growth, or in case of neurological deterioration, treatment with microsurgery (MS) or radiosurgery is considered. MS is the method of choice for large tumors with radiological or neurological signs of brainstem compression. Nowadays, MS can be performed with low mortality, but it is still associated with a significant risk of neurological sequelae such as hearing loss and facial nerve palsy [3, 5, 7, 8]. Single-dose stereotactic radiosurgery (SRS) and fractionated stereotactic radiotherapy (FSRT) aiming to arrest tumor growth are the most commonly used noninvasive alternatives. In case series of patients suitable for radiosurgery/radiotherapy, the progression-free survival rates are comparable to those of case series of microsurgical resection of VS [14, 16, 30, 53]. Further, controlled studies and subsequent systematic reviews support the use of radiosurgery in VS patients eligible for both surgery and radiosurgery [23, 36, 38, 42, 43, 46]. Additionally, SRS and FSRT entail a lower incidence of side effects such as facial nerve palsy and hearing deterioration [9, 47]. In a recent Cochrane review [35] of controlled randomized trials, the authors concluded that there was insufficient evidence to recommend either surgical or radiation therapy in the treatment of VS. However, six prospective intervention studies comparing MS to SRS all concluded that SRS demonstrates similar effectiveness in terms of progression-free survival and that SRS demonstrates a significantly lower risk of neurological deteriorations such as facial nerve palsies [23, 36, 38, 42, 43, 46]. Results of these studies were analyzed in a systematic review comparing Gamma Knife radiosurgery with microsurgical resection in VS eligible for both treatments and demonstrated better outcome after Gamma Knife radiosurgery [40, 55].

There are some different advantages/disadvantages related to the radiation techniques in SRS and FSRT. Linear accelerator (LINAC)-based systems for FSRT are more available, and due to the low fractionation dose, larger tumors can be treated. The theoretical rationale for dividing the prescribed total dose into 25–30 fractions is mainly to increase the chance of targeting the tumor cells in the most radiation-sensitive phase of the cell cycle [41]. On the contrary, SRS relies on a single high radiation dose, potentially affecting tumor cells also in the non-dividing phase. Physical properties of Gamma Knife SRS allow a steep radiation gradient on the tumor margin, which is important for keeping the dose to adjacent structures low. While it is known that some tumors resistant to fractionated radiotherapy may responds to radiosurgery, the precise differences of the radiobiological properties of the two modalities are to some extent still an issue of discussion [25]. With the evolution of hypfractionated regimes a continuum has also emerged between radiotherapy and radiosurgery, and sometimes the distinction might be unclear. The most widely accepted definition of what constitutes radiosurgery seems to be that of the AANS/CNS, defining radiosurgery as a maximum of five treatment sessions (however typically performed in a single session) [4].

A stereotactic frame is always used for Gamma Knife SRS, which further enhances the precision of the delivered radiation, and no margin is needed to compensate for movement of the head [54]. However, this set-up may be perceived as more cumbersome and invasive. There is no consensus in the literature on the definition of “stereotactic” fractionated radiotherapy. However, all reports concerning FSRT subsequently included in this review utilized LINAC-based systems using either face masks or relocatable frames for fixation, and all used this terminology, which is why this has also been adopted for this review.

Since there is a lack of high evidence studies comparing the different radiation modalities, local treatment policies and availability have determined the choice of irradiation modality. In the present systematic review, we aim to study the effectiveness and safety of SRS compared to FSRT for the treatment of unilateral VS.

## Methods

The study was carried out based on the recommendations outlined in the Cochrane Handbook for Systematic Reviews of Interventions [20]. The review protocol was conducted according to the PRISMA statement [34] and registered in PROSPERO International Prospective Register of Systematic Reviews (registration no. CRD 42015029505). Five databases—EMBASE, MEDLINE, Web of Science, clinicaltrials.gov and the Cochrane library—were systematically searched for records concerning SRS and/or FSRT for the treatment of VS published between January 1995 and December 2014. The systematic search was executed with Medical Subject Headings (MeSH) terms if available or as free text. The searches were performed from January 28–February 2 2015. The specific search criteria are available in Supplementary Table S1.

An initial review of eligibility and relevance was performed based on abstracts for all records by the senior author (PF). In the second round all remaining records were review according to a Systematic Review Form (SRF) by two authors independently (PF and OP, or JB and NBS). Reference lists of the reviewed papers were crosschecked for further publications of relevance. Statistical calculations were performed using the MedCalc tool (www.medcalc.org/calc).

End points were dichotomous and are presented as frequencies and odds ratios (OR) with 95% confidence intervals (CI). Regular meta-analyses were not attempted for uncontrolled case series. Results were instead reported as summarized frequencies for the individual end points without considering heterogeneity between studies.

### Inclusion and exclusion criteria

Studies on adults (≥18 years) including patients with a minimal median (if the median was not reported, mean was used) follow-up of 5 years, with unilateral VS (diagnosed with histopathology or typical MR appearance) published in the period 1995–2014 and treated with either SRS or FSRT were included.

Included studies were categorized according to the following: S1, randomized controlled trials (RCTs), quasi-RCTs of any sample size; S2, quantitative comparative study designs not being prospective randomized studies, but including cohort studies and case control studies with ≥50 patients in total with a median follow-up time of >5 years; S3, case series with ≥50 patients and a median follow-up time of >5 years.

Papers concerning patient cohorts treated with SRS or FSRT for novel tumors or regrowth of a previously operated tumor were included. Patient cohorts receiving combined treatment (i.e., surgery + SRS/FSRT) for novel tumors were excluded.

Patient cohorts including patients with neurofibromatosis type 2 (NF2) were excluded unless the NF2 patients could be specified and excluded from the study cohort and result data or the ratio of NF2 patients in the cohort was so small that any influence on the overall outcome data of the cohort was considered marginal.

When more than one publication was found reporting data from the same or partly overlapping patient cohorts, we reserved the right to choose the largest, most recent or the most suitable publication for the purpose of this review for inclusion. Other papers reporting data from the same patient cohort were excluded.

### Primary end point

Primary end point was loss of tumor control defined as frequency of patients requiring a new VS-targeted intervention (i.e., re-treatment with SRS, FSRT or surgery).

### Secondary end points

Secondary end points were: frequency of death due to tumor progression; frequency of VS patients with functional hearing [Gardner Roberson (GR) class 1 and 2] deteriorating to non-serviceable hearing (GR class 3–5); frequency of loss of tumor control as defined by the authors; frequency of patients with deterioration of facial nerve function after treatment; frequency of patients with deterioration of trigeminal nerve function after treatment.

## Results

### Literature search

**Fig. 1:**
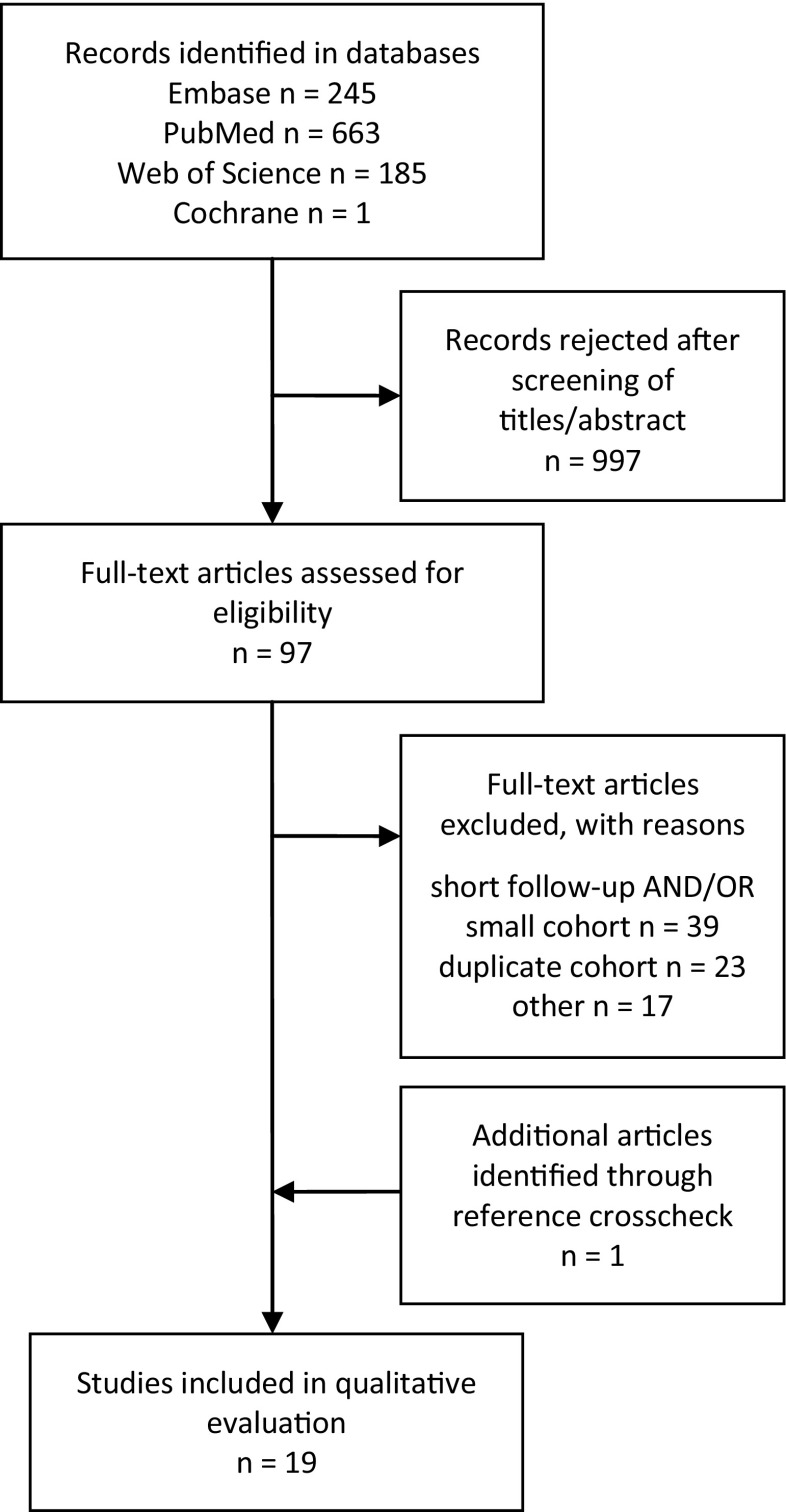
Schematic overview of the number of identified records for the systematic steps of the review process. In total 19 papers fulfilled the inclusion criteria with regard to definition of the primary end point, follow-up and size of the patient cohort

The initial literature search yielded 1094 records after duplicate results had been eliminated (Fig. 1 and Supplementary Table S1). The initial review could discard 997 records as not relevant to the topic or as inadequate regarding cohort size or follow-up time, and after reference cross-check 98 records were reviewed according to a systematic review protocol. After the second review round a final 19 records were found to fulfill all inclusion criteria [2, 6, 10, 15, 17, 19, 22, 24, 28, 29, 33, 36, 39, 44, 48, 50, 52, 53, 56]. No randomized studies (S1) were identified on the subject. A number of non-randomized quantitative comparative studies (S2) were found; however, none of them fulfilled all criteria regarding follow-up time and/or defining the primary outcome parameter.

### Tumor control

**Fig. 2:**
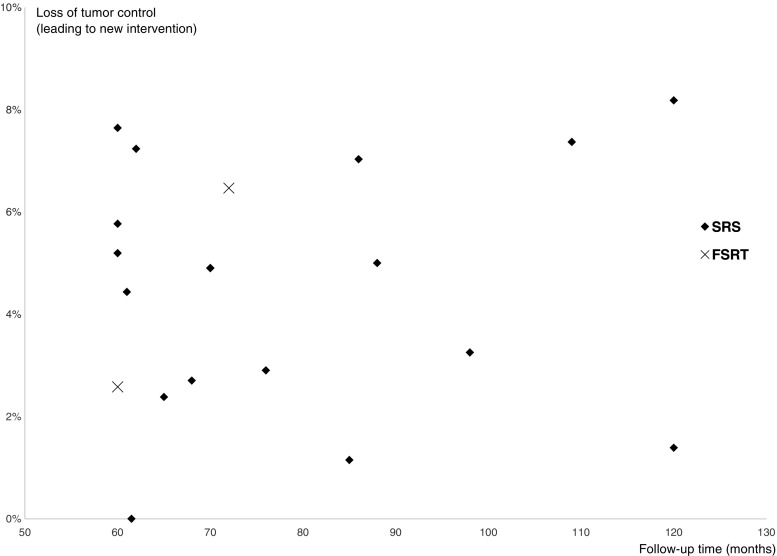
Loss of tumor control distributed according to median follow-up time for the included studies. No apparent trend for increasing failure was noted with longer follow-up times after 5 years

Two case series reported the outcome data after FSRT treatment and 17 after SRS treatment. The average loss of tumor control leading to a new VS-targeted intervention was 4.8% for FSRT and 5.0% for SRS within the follow-up period (Table 1). The tumor volumes/sizes were equal for the SRS and FSRT studies. All SRS studies had a median marginal dose of 12–13 Gy, and both FSRT studies had a median total dose of 50 Gy in fractions of 1.8–2 Gy (Supplementary Table S2). There was no apparent trend for loss of tumor control in relation to median follow-up times (Fig. 2). Since these were all uncontrolled cohort reports (i.e., S3 studies), no statistical meta-analysis was attempted. Potential reporting bias was assessed using a funnel plot for the SRS case series (Supplementary Fig. S1) and revealed approximately equal distribution of treatment failure leading to a new intervention, independent of study size.

**Table 1:** Loss of tumor control ratios for the respective studies defined as need for a new targeted treatment against the vestibular schwannoma

Author (year)	Treatment failure (leading to a new intervention)	
	SRS	FSRT
Unger et al. [52]	3/60	
Iwai et al. [22]	3/52	
Myrseth et al. [36]	5/102	
Hempel et al. [19]	4/123	
Liu et al. [29]	2/74	
Chopra et al. [10]	3/216	
Fukuoka et al. [15]	12/157	
Pollock et al. [44]	13/293	
Nagano et al. [39]	1/87	
Roos et al. [48]	2/84	
Sun et al. [50]	14/190	
Yomo et al. [56]	8/154	
Hasegawa et al. [16, 17]	36/440	
Kim et al. [24]	0/60	
Boari et al. [6]	11/379	
Mindermann et al. [33]	17/235	
Wangerid et al. [53]	9/128	
Aoyama et al. [2]		13/201
Litre et al. [28]		4/155
Total	143/2834 (5.0%)	17/356 (4.8%)

Loss of tumor control as defined by author (i.e., radiological progression) showed similar results such as loss of control leading to a new intervention (Supplementary Table S3). Only one study reported any mortality due to tumor progression [17], in 4 of 440 patients.

### Hearing deterioration

**Fig. 3:**
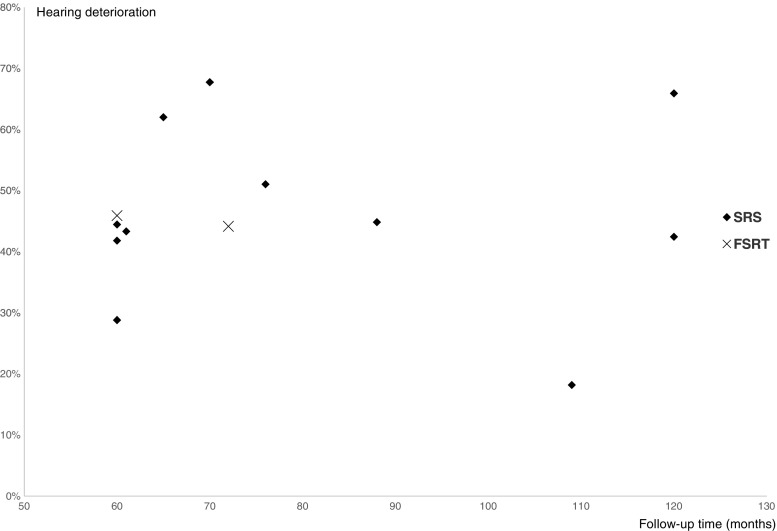
Hearing deterioration ratios distributed according to median follow-up time for the included studies. No apparent trend for increasing deterioration ratios was noted with longer follow-up times after 5 years

Hearing deterioration after treatment was defined as deterioration from serviceable hearing (Gardner-Robertson I or II) to non-serviceable hearing (Gardner-Robertson III-V) on the treated side. Quantitative hearing data were available for 13 of the included studies (11 SRS, 2 FSRT). In total 52% of the SRS patients had serviceable hearing before treatment vs. 59% of the FSRT patients. The average deterioration ratio for patients with serviceable hearing before treatment was 49% for SRS and 45% for FSRT (Table 2). No apparent trend for increasing hearing deterioration was discernable in relation to median follow-up times (Fig. 3).

**Table 2:** Hearing deterioration for the 14 included studies with quantitative hearing data, defined as deterioration from serviceable (Gardner-Robertson I and II) to non-serviceable (Gardner-Robertson III–V) hearing

Author (year)	Gardner Robertson I+II		Ratio deteriorated SRS	Gardner Robertson I+II		Ratio deteriorated FSRT
	Before SRS	After SRS		Before FSRT	After FSRT	
Unger et al. [52]	29/29	16/29	13/29			
Iwai et al. [22]	18/47	10/47	8/18			
Myrseth et al. [36]	31/60	10/60	21/31			
Chopra et al. [10]	106/162	61/162	45/106			
Fukuoka et al. [15]	59/152	42/152	17/59			
Roos et al. [48]	50/91	19/91	31/50			
Sun et al. [50]	22/190	18/190	4/22			
Yomo et al. [56]	110/154	64/154	46/110			
Hasegawa et al. [16, 17]	135/345	46/345	46/135			
Kim et al. [24]	60/60	34/60	26/60			
Boari et al. [6]	96/96	47/96	47/96			
Aoyama et al. [2]				77/77	43/77	34/77
Litre et al. [28]				61/158	33/158	28/61
Total			349/716 (49%)			62/138 (45%)

### Facial and trigeminal nerve deterioration

Facial and trigeminal nerve deterioration was defined as any new onset or worsening of previous facial paralysis, facial spasm, facial tingling or loss of sensation on the treated side—either transient or permanent. Fourteen of the included studies (12 SRS, 2 FSRT) reported quantitative data for cranial nerve deterioration. The facial nerve deterioration was 3.6% for SRS and 11.2% for FSRT (Table 3), and the trigeminal nerve deterioration was 6.0% for SRS and 8.4% for FSRT (Table 4).

**Table 3:** Facial nerve deterioration defined as any transient or permanent impairment of facial nerve function, either new or worsening of preexisting symptoms

Author (year)	Facial nerve deterioration	
	SRS	FSRT
Unger et al. [52]	5/60	
Iwai et al. [22]	3/52	
Hempel et al. [19]	0/123	
Liu et al. [29]	3/63	
Chopra et al. [10]	0/216	
Fukuoka et al. [15]	2/157	
Roos et al. [48]	9/102	
Sun et al. [50]	28/190	
Yomo et al. [56]	1/154	
Hasegawa et al. [16, 17]	7/440	
Boari et al. [6]	11/379	
Wangerid et al. [53]	5/128	
Aoyama et al. [2]		19/201
Litre et al. [28]		21/155
Total	74/2064 (3.6%)	40/356 (11.2%)

**Table 4:** Trigeminal nerve deterioration defined as any transient or permanent impairment of trigeminal nerve function, either novel or worsening of preexisting symptoms

Author (year)	Trigeminal nerve deterioration	
	SRS	FSRT
Unger et al. [52]	3/60	
Iwai et al. [22]	2/52	
Hempel et al. [19]	7/121	
Liu et al. [29]	5/74	
Chopra et al. [10]	8/216	
Fukuoka et al. [15]	7/159	
Roos et al. [48]	15/102	
Sun et al. [50]	44/190	
Yomo et al. [56]	2/154	
Hasegawa et al. [16, 17]	3/440	
Boari et al. [6]	26/379	
Wangerid et al. [53]	3/128	
Aoyama et al. [2]		23/201
Litre et al. [28]		7/155
Total	125/2075 (6.0%)	30/356 (8.4%)

### Post hoc subanalysis of case-control (S2) studies not fulfilling inclusion criteria

**Fig. 4:**
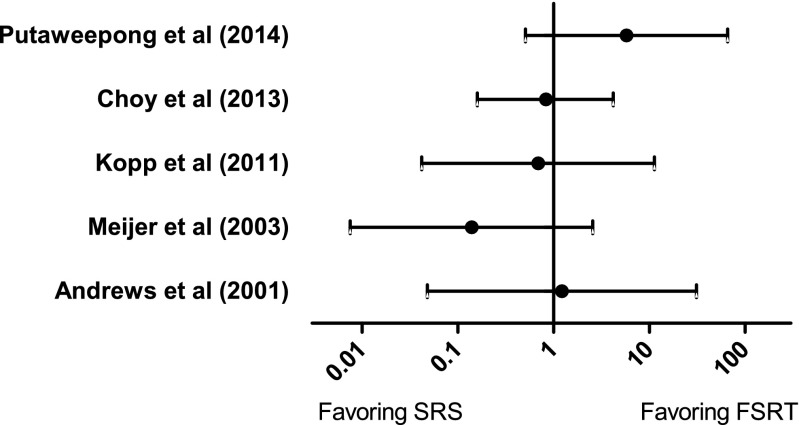
Odds ratios with 95% CI for five single-center comparative studies comparing loss of tumor control after SRS compared to FSRT

A number of non-randomized quantitative comparative studies (S2) were identified in the literature search. Although none of these fulfilled all inclusion criteria with regard to follow-up time and definition of the primary end point to be included in the primary analysis, five of these still reported quantitative data on loss of tumor control (as defined by the author). Since these reports contain comparative data for SRS and FSRT from single centers, a separate post-hoc subanalysis of these studies was still undertaken. None of these studies showed any significant difference in tumor control between SRS and FSRT (Fig. 4).

## Discussion

In the present systematic review, we aimed to investigate the current scientific support for the long-term effectiveness and safety of SRS compared to FSRT for the treatment of unilateral VS. Nineteen case series met the inclusion criteria (17 STS and 2 FSRT) and were enrolled in the systematic review. The primary end point was loss of tumor control necessitating a new VS-targeted intervention and showed comparable results for SRS (mean 5.0%) and FSRT (mean 4.8%) in the included case series. Similar results were found when analyzing tumor control ratios as defined by the different authors regardless of the need for a new intervention. Thus, the current data provide no indication of one treatment technique being superior to the other with regard to tumor control. A caveat when analyzing these results is that only two FSRT studies (reporting on a total of 356 patients) met the inclusion criteria and also that these studies had a shorter follow-up time compared to several of the SRS studies (reporting on a total of 2834 patients). This substantial gap in reported follow-up time between centers using SRS or FSRT could merely represent different follow-up traditions, but certainly indicates a risk of detection bias in the FSRT group. One additional study [51] was identified in the initial literature search reporting on hypofractionated radiotherapy (HfRT) for VS using the CyberKnife (18 Gy in 3 fractions). However, since HfRT from a radiobiologic point of view is likely to be mechanistically more similar to SRS than to conventional FSRT, this study was not included in the FSRT group. This study showed a favorable outcome with no need for reintervention in any of the 117 treated patients within the 61-month median follow-up. Although promising, these results from a single study would need to be additionally confirmed.

We found no randomized controlled studies comparing SRS and FSRT for vestibular schwannomas. One study initially attempted a randomized design [1], which had to be abandoned because of patient expectations or physician’s bias. Another study had a degree of “pseudo-randomization” based on the dentate status, where patients were assigned to the different treatment arms based on whether they could be reliably and reproducibly fixated for FSRT or not [32]. However, both of these studies were excluded from the main analysis because of the too short follow-up time. Also no other case-control studies comparing SRS and FSRT were found to be eligible according to the inclusion criteria in the present systematic review. These studies were all either too small (<50 patients), reported too short follow-up times (<5 years) or did not report on the primary end point (risk for a new intervention) in a quantifiable manner. Some studies also allocated patients differently to SRS or FSRT treatment based on tumor size and pretreatment hearing function, creating unbalanced groups with respect to selection bias [11–13]. However, since these are likely the most “comparable” groups available in the literature, a separate post hoc analysis of comparative studies where quantitative data could be extracted was undertaken (Fig. 4), but showed no differences between SRS and FSRT with regard to tumor control. These results are also in line with a recent large German multicenter report on long-term (median 67 months) follow-up after radiation therapy of VS [14], which showed equal tumor control ratios for SRS and FSRT (this paper could not be included in the statistical analysis because of lack of quantifiable outcome data).

As for all systematic reviews, the quality of the included studies is the limiting factor. When analyzing results of treatment efficacy for benign tumors, long-term follow-up is of essential importance. In the case of vestibular schwannomas, tumor control on the order of up to 70% after 1–3 years can also be expected with conservative management [49]. Remarkably many reports had to be excluded from this review because of the short follow-up time. Complications, such as cranial nerve radiation toxicity and hearing deterioration, usually emerge within the first years after treatment, and these safety end points are therefore less sensitive to short follow-up times.

To be certain of a robust primary end point that was independent of subjective evaluation and did not differ substantially between centers, we used the need for a new intervention instead of radiological loss of tumor control. Progression necessitating a new intervention is also the most clinically relevant end point, since an increase in tumor volume (pseudoprogression) after radiation therapy is common and is seldom symptomatic [18]. However, several of the reviewed articles reported only on radiological tumor progression, without any data on the need for new treatments in these patients and were therefore excluded from this review. Furthermore, studies using a dichotomous outcome measure, such as the need for a new intervention, also need to be powered enough to detect these rare events. For this reason, studies reporting fewer than 50 patients were excluded. Multiple studies also had to be excluded because of reporting data from the same or partly overlapping patient cohorts. Redundant publishing of patient data constitutes a problematic issue in the context of systematic reviews, since this is rarely cross-referenced in the reports and thus increases the risk of duplicate publication bias [20].

One of the major benefits of FSRT or SRS in comparison to MS for VS is the decreased risk of cranial nerve damage. There was a small difference in patients with serviceable hearing before treatment between groups (SRS 52% vs. FSRT 59%). This may reflect the tendency in some centers to refer patients with serviceable hearing to FSRT [11–13]. The hearing deterioration showed no substantial difference between the FSRT group (45%) compared to SRS (49%). Since these data are based on uncontrolled case series, no analysis of statistical significance was undertaken.

Contrary to what has previously been reported in some single-center comparative studies [11, 26, 32], an increased risk of facial and trigeminal nerve deterioration was found in the included FRST studies compared to studies on SRS (Tables 3 and 4). For the purpose of this review, all reported symptoms of nerve deterioration—mild and transient as well as permanent—were included in the analysis.

## Conclusion

We identified several studies reporting the long-term tumor control rate after SRS, while only two studies reported on long-term tumor control after FSRT could be identified, engendering a more robust support for favorable long-term tumor control with SRS. The risk for facial and trigeminal nerve deterioration was less for patients treated in the SRS series compared to VS patients receiving FSRT, while the chance of preserved hearing showed no difference between the two treatment groups. To establish guidelines for radiotherapeutic treatment in vestibular schwannomas, a RCT or a prospective controlled study comparing SRS and FSRT would be needed. While awaiting results from such a study, this review reveals a need for FSRT case series with larger cohorts and longer follow-up tim to obtain more solid data on long-term tumor control for this treatment modality.

## Electronic supplementary material

Fig. S1 (PDF 35 kb)

Table S1 (PDF 24 kb)

Table S2 (PDF 25 kb)

Table S3 (PDF 25 kb)
