# Supplementary figures and images for: Stereotactic radiosurgery vs. fractionated radiotherapy for tumor control in vestibular schwannoma patients: a systematic review
Source: Acta Neurochir (Wien). 2017 Apr 13;159(6):1013–21. doi: 10.1007/s00701-017-3164-6 (PMC5425507; doi:10.1007/s00701-017-3164-6)

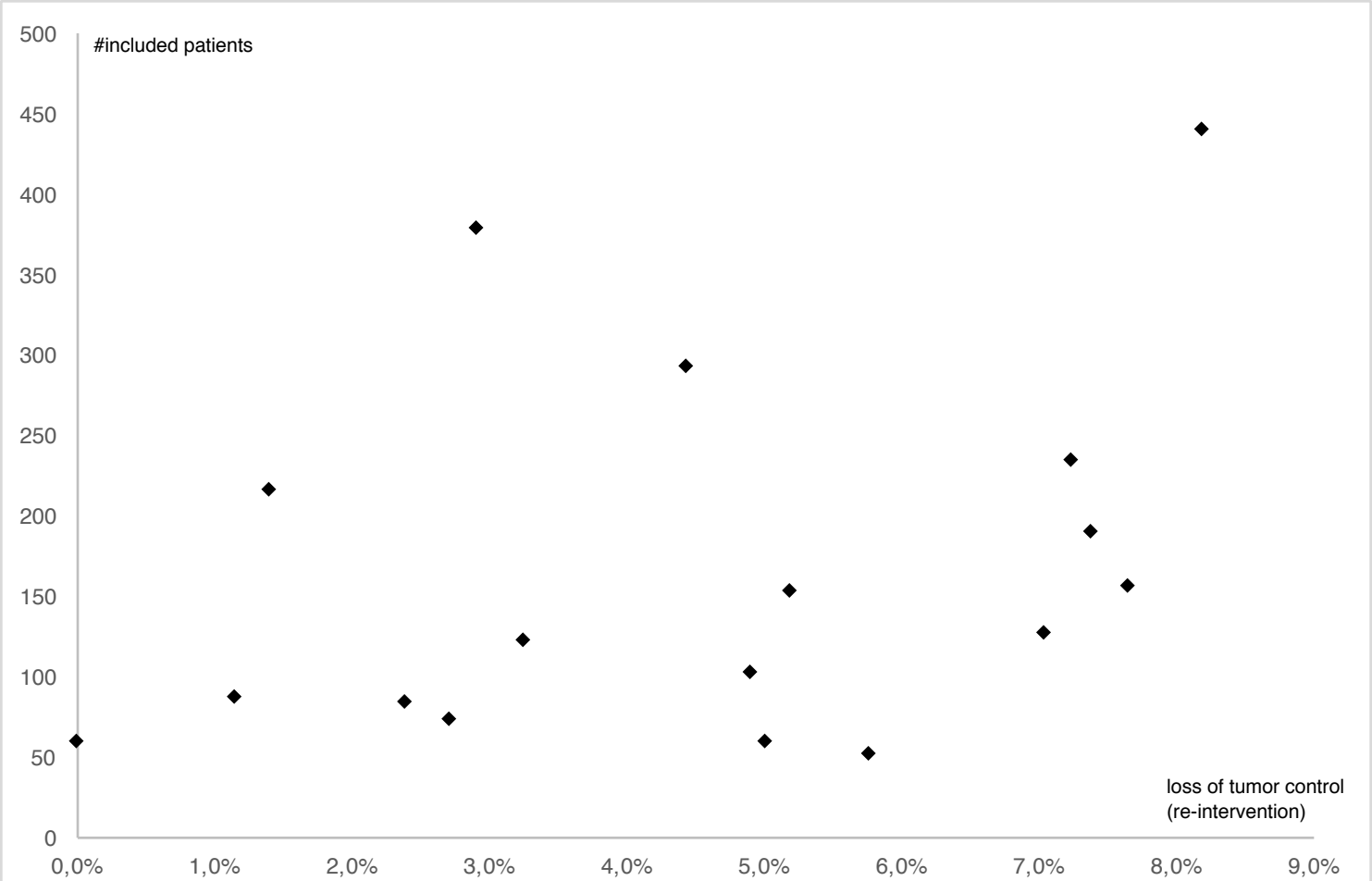

Supplement: Supplementary file 1 — (PDF 35 kb) [file 701_2017_3164_MOESM1_ESM.pdf]
