# Supplementary material for: Stereotactic radiosurgery vs. fractionated radiotherapy for tumor control in vestibular schwannoma patients: a systematic review
Source: Acta Neurochir (Wien). 2017 Apr 13;159(6):1013–21. doi: 10.1007/s00701-017-3164-6 (PMC5425507; doi:10.1007/s00701-017-3164-6)
Supplement: Supplementary file 2 — (PDF 24 kb) [file 701_2017_3164_MOESM2_ESM.pdf]

Web of Science 15-01-28

Nr		Results
1	neuroma, acoustic OR vestibular schwannoma (topic)	11923
2	radiosurgery OR stereotactic radiosurgery OR fractionated radiosurgery OR gamma knife radiosurgery (topic)	24929
3	radiotherapy OR fractionated radiotherapy OR external beam radiotherapy OR radiation therapy (topic)	484461
4	# 1 AND # 2 AND # 3	771
5	Publ datum 1995-2014, article, review, other, abstract, clinical trial, språk eng, fra, spa, ty	531

Cochrane 15-01-28

Nr		Results
1	neuroma, acoustic OR vestibular schwannoma (ti, ab, kw)	96
2	radiosurgery (ti, ab, kw)	317
3	radiotherapy (ti, ab, kw)	12499
4	# 1 AND # 2 AND # 3	4<br>DARE/1<br>CENTRAL/3

Embase 15-02-02

Nr		Results
1	acoustic AND (neurinoma/exp OR neurinoma) (emtree)	7328
2	vestibular AND (schwannoma/exp OR schwannoma)	2872
3	# 1 OR # 2	8451
4	radiosurgery/exp OR radiosurgery (emtree)	39036
5	radiotherapy/exp OR radiotherapy (emtree)	533776
6	# 3 AND # 4 AND # 5	875
7	Publ datum 1995-2014, abstract tillgängligt, språk eng, fra, spa, ty	676
