# Supplementary material for: Stereotactic radiosurgery vs. fractionated radiotherapy for tumor control in vestibular schwannoma patients: a systematic review
Source: Acta Neurochir (Wien). 2017 Apr 13;159(6):1013–21. doi: 10.1007/s00701-017-3164-6 (PMC5425507; doi:10.1007/s00701-017-3164-6)
Supplement: Supplementary file 3 — (PDF 25 kb) [file 701_2017_3164_MOESM3_ESM.pdf]

**Author (*year*)**	**Tumor volume (ml)**	**Max diameter (mm)**	**Margin dose (Gy)**	**Isodose (%)**	**Max dose (Gy)**	**Total dose (Gy)**	**Dose (Gy)/Fractionation**
Unger *et al.* (2002)	3.8 (0.02-27.22)		12 Gy (9-18Gy)	30-80%			
Iwai *et al.* (2003)	3.6 (0.7-24.9)		12 Gy (8-12)				
Myrseth *et al.* (2005)			12.2 Gy (10-20)		35.3 Gy (17-40)		
Hempel *et al.* (2006)	1.6 (0.1-9.9)		13 Gy (10-14.5)	40-85%	22.7 (15.6-32.5)		
Liu *et al.* (2006)	10.8 (0.11-27.8)		12.3 (10-14)		24.9 (21-30)		
Chopra *et al.* (2007)	1.3 (0.08-37.5)		13 (12-13)	50-65	26 (20-26)		
Fukuoka *et al.* (2009)	2.0 (0.1-18.6)		12.0 (9-15)		24 (17.1-30)		
Pollock *et al.* (2009)	2.9		13 (12-14)		28 (24-30)		
Nagano *et al.* (2010)	2.5 (0.1-13.2)		12.0 (10.5-13.0)	50 (50-67)			
Roos *et al.* (2011)		22 (11-40)	12 (12-14)				
Sun *et al.* (2012)	3.6 (0.3-27.3)		13.0 (6.0-14.4)	45 (20-68)	28.0 (15.0-60.0)		
Yomo *et al* . (2012)	0.73 (0.03-5.37)		12.1 (9-14)	51.1 (40-80)			
Hasegawa *et al.* (2013)	2.8 (0.07-36.7)		12.8 (10-18)	50 (40-95)	25 (13-36)		
Kim *et al.* (2013)	0.34 (0.03-1.00)		12.2 (11.5-13.0)	51 (50-70)	24.3 (17.1-26.2)		
Boari *et al* . (2014)	1.2 (0.013-14.3)		13 (11-15)	50 (40-63)	26 (20-32.6)		
Mindermann *et al.* (2014)	1.85		12.9	50	25.8		
Wangerid *et al.* (2014)	1.65 (0.1-8.4)		12.3 (11-16)	50-60	26 (17-47)		
Aoyama *et al.* (2012)		20 (7-48)		80-90		50 (30-50)	2 (1.8-2)*****
Litre *et al.* (2013)	2.7 (1.5-15.5)					50	1.8

Dose schedules of the studies included in the analysis. Values are reported as median or mean (range).

* 7 of 201 patients in *Aoyama et al.* recieved a hypfractionated treatment regime.
