# Supplementary material for: Stereotactic radiosurgery vs. fractionated radiotherapy for tumor control in vestibular schwannoma patients: a systematic review
Source: Acta Neurochir (Wien). 2017 Apr 13;159(6):1013–21. doi: 10.1007/s00701-017-3164-6 (PMC5425507; doi:10.1007/s00701-017-3164-6)
Supplement: Supplementary file 4 — (PDF 25 kb) [file 701_2017_3164_MOESM4_ESM.pdf]

	Loss of tumor control (as defined by author)	
**Artikel**	**SRS**	**FSRT**
Unger *et al.* (2002)	4/60	
Iwai *et al.* (2003)	3/52	
Myrseth *et al.* (2005)	11/102	
Hempel *et al.* (2006)	5/123	
Liu *et al.* (2006)	3/74	
Chopra *et al.* (2007)	7/216	
Fukuoka *et al.* (2009)	12/157	
Pollock *et al.* (2009)	15/293	
Nagano *et al.* (2010)	7/87	
Roos *et al.* (2011)	2/84	
Sun *et al.* (2012)	20/190	
Yomo *et al* . (2012)	8/154	
Hasegawa *et al.* (2013)	31/440	
Kim *et al.* (2013)	0/60	
Boari *et al* . (2014)	11/379	
Mindermann *et al.* (2014)	21/235	
Wangerid *et al.* (2014)	10/128	
Aoyama *et al.* (2012)		13/201
Litre *et al.* (2013)		4/155
**Total**	**6.2%**	**4.8%**
